# The High Burden of Tuberculosis (TB) and Human Immunodeficiency Virus (HIV) in a Large Zambian Prison: A Public Health Alert

**DOI:** 10.1371/journal.pone.0067338

**Published:** 2013-08-14

**Authors:** German Henostroza, Stephanie M. Topp, Sisa Hatwiinda, Katie R. Maggard, Winifreda Phiri, Jennifer B. Harris, Annika Krüüner, Nathan Kapata, Helen Ayles, Chisela Chileshe, Stewart E. Reid

**Affiliations:** 1 Department of Medicine, University of Alabama at Birmingham, Birmingham, Alabama, United States of America; 2 Centre for Infectious Disease Research in Zambia, Lusaka, Zambia; 3 Department of Obstetrics and Gynecology, University of North Carolina at Chapel Hill, Chapel Hill, North Carolina, United States of America; 4 National Tuberculosis Program, Ministry of Health, Lusaka, Zambia; 5 Department of Clinical Research, London School of Hygiene & Tropical Medicine & Zambia AIDS Related Tuberculosis Project, Lusaka, Zambia; 6 Prisons Health Service, Ministry of Home Affairs, Lusaka, Zambia; 7 Department of Medicine, University of North Carolina at Chapel Hill, Chapel Hill, North Carolina, United States of America; Institute of Infectious Diseases and Molecular Medicine, South Africa

## Abstract

**Background:**

Tuberculosis (TB) and human immunodeficiency virus (HIV) represent two of the greatest health threats in African prisons. In 2010, collaboration between the Centre for Infectious Disease Research in Zambia, the Zambia Prisons Service, and the National TB Program established a TB and HIV screening program in six Zambian prisons. We report data on the prevalence of TB and HIV in one of the largest facilities: Lusaka Central Prison.

**Methods:**

Between November 2010 and April 2011, we assessed the prevalence of TB and HIV amongst inmates entering, residing, and exiting the prison, as well as in the surrounding community. The screening protocol included complete history and physical exam, digital radiography, opt-out HIV counseling and testing, sputum smear and culture. A TB case was defined as either bacteriologically confirmed or clinically diagnosed.

**Results:**

A total of 2323 participants completed screening. A majority (88%) were male, median age 31 years and body mass index 21.9. TB symptoms were found in 1430 (62%). TB was diagnosed in 176 (7.6%) individuals and 52 people were already on TB treatment at time of screening. TB was bacteriologically confirmed in 88 cases (3.8%) and clinically diagnosed in 88 cases (3.8%). Confirmed TB at entry and exit interventions were 4.6% and 5.3% respectively. Smear was positive in only 25% (n = 22) of bacteriologically confirmed cases. HIV prevalence among inmates currently residing in prison was 27.4%.

**Conclusion:**

Ineffective TB and HIV screening programs deter successful disease control strategies in prison facilities and their surrounding communities. We found rates of TB and HIV in Lusaka Central Prison that are substantially higher than the Zambian average, with a trend towards concentration and potential transmission of both diseases within the facility and to the general population. Investment in institutional and criminal justice reform as well as prison-specific health systems is urgently required.

## Introduction

The Global Plan to Stop TB 2011–2015, launched in 2010 has an overall aim to halve TB mortality and prevalence rates by 2015, compared with a 1990 baseline. Specific objectives of this plan include ensuring early diagnosis of all TB cases, including amongst vulnerable populations such as prisoners [1].

The poor health and living conditions that facilitate transmission of tuberculosis and HIV in prisons are now widely acknowledged to constitute a violation of human rights and a public health threat [2]. Estimates of prison-related TB rates range from 5 to 50 times higher than those in the general community in both lower and middle income countries (LMIC) and industrialized nations [3]–[7]. Despite the implications of such projections for population health, there remain many gaps in our knowledge of prison-related burden of disease. Due to the relatively few studies conducted in this field, our understanding of TB and HIV rates in prisons as compared to the wider community are often based on estimates. In sub-Saharan Africa in particular, the true prevalence of TB and HIV in prisons is often unknown, much less the proportion of disease acquired in prison. Meanwhile, the prison conditions in many, if not most prisons in the region provide near-perfect conditions for the spread of both HIV and TB [2].

In Zambia, despite progress in TB control and HIV treatment in public sector health facilities, high rates of disease persist. TB prevalence (all forms) in the general population is estimated at 352/100,000 [8]. However, a study in two communities of Lusaka province found the prevalence of bacteriologically confirmed TB to be much higher at 870/100,000 [9]. Nationwide HIV prevalence is estimated to be 12.5% [10] while in Lusaka province it is 21% [11]. Limited evidence exists for TB or HIV prevalence in the country's prisons with the most recent published studies being conducted more than ten years ago; these reported an HIV prevalence of 27% [12] and a conservative estimate for TB prevalence of 4%, or 4000/100,000 [13]; both of these estimates were substantially higher than national estimates at the time of the studies.

A 2010 Human Rights Watch Report outlined conditions in Zambian prisons including poor environmental, physical and emotional circumstances in which inmates live and highlighting the substantial risk these posed to inmate health [14]. That same year, the Zambia Prisons Service (ZPS) and Zambian Ministry of Health (MOH), in partnership with the Centre for Infectious Disease Research in Zambia (CIDRZ) and the Zambia AIDS Related TB Project (ZAMBART) obtained funds through the TB REACH initiative of the Stop TB Partnership to establish a TB and HIV screening program in six major prisons across three provinces. An operations research component was included as part of the program, to facilitate monitoring and evaluation and enable the prevalence of TB and HIV in prisons to be reported to policy makers and public health authorities. This paper reports data on the prevalence of TB and HIV amongst inmates entering, residing within and exiting the largest of those facilities – Lusaka Central Prison.

## Methods

### Ethics Statement

The protocol was approved by the biomedical research ethics committee of the University of Zambia (001-03-11), and the institutional review board of the University of Alabama at Birmingham (F101014011), United States of America. Both institutions waived informed consent for participation in screening activities since they were considered standard of care. Special attention was paid to the vulnerable nature of this population in the context of provider initiated HIV testing services. All HIV counseling was conducted by experienced psycho-social counselors in a private one-on-one setting. Inmates had the chance to opt-out of HIV testing, or to choose not to receive the results if they did test. No inmate was required to carry any form or indication of results that may later identify him or her as HIV positive.

### Setting

Lusaka Central Prison is a medium security facility, built in the 1923 by the British administration of then Northern Rhodesia. Capacity of the facility was established at 200 inmates and its location was on the outskirts of the recently established town of Lusaka. By 2010, Lusaka Central Prison was housing between 1400 and 1500 inmates without any upgrade in infrastructure. In modern-day Lusaka, the prison is located at the center of a densely populated services area, which includes Zambia's University Teaching Hospital, and is adjacent to several low-income residential areas or ‘compounds’. Inmates are a mixture of convicted prisoners and remandees awaiting trial, with resultant high turnover.

### Intervention

Between November 2010 and April 2011 a TB and HIV screening program was established in Lusaka Central prison. Prior to the inception of the TB REACH program, a ZPS employed clinical officer (CO) nominally provided a health review for incoming inmates. However, human resource shortages meant that reviews were cursory at best. The overall goal of the TB REACH program was to develop systems and capacity to ensure that TB and HIV screening were conducted for all inmates coming into the facility, and that diagnosed cases received TB and/or HIV treatment through established treatment programs available within the prison or in neighboring clinics. The intervention focused on building and/or upgrading infrastructure, training staff to follow routine screening and diagnostic protocols for TB and HIV, and training a cadre of inmate peer educators to assist with information dissemination and case-finding.

The primary purpose of the screening program was to detect and treat undiagnosed cases residing within the prison. Additional objectives were to understand the prevalence of TB and HIV in the facility and the surrounding prison camp community and determine whether rates of disease were higher amongst exiting inmates compared to entering inmates. We first screened a consecutive sample of inmates exiting the prison (‘exit’ screening) in order to capture the proportion with active TB released into the community without the influence of the screening intervention. We then conducted a comprehensive screening of all inmates entering (‘entry’ screening) and residing within the prison (‘mass’ screening). Finally, we conducted a community screening for prison staff and their families in the surrounding prison camp. All individuals diagnosed with TB and/or HIV were referred to the closest MOH health clinic for treatment. Table 1 lists the screening activities in chronological order.

**Table 1:** Schedule of Screening Activities.

Screening Phase	Population	Duration
Exit Screening	All inmates prior to release from the prison to the general community	November–February 2011
Mass Screening	All inmates currently residing within the prison	January–April 2011
Entry Screening	All new inmates entering the prison	February–April 2011
Community Screening	Prison staff and their families living in the prison camp community surrounding the prison	April 2011

### TB and HIV Screening Protocol

Entry and mass screening was an ongoing, two-day process. Each day, prison management and inmate peer educators produced lists of inmates to be screened and collected two spot sputum samples from inmates irrespective of the presence of symptoms. After providing sputum samples, inmates were referred to a nurse or inmate peer educator for TB risk factor assessment and symptom screening. Inmates were subsequently offered provider initiated HIV testing and counseling (PITC) by a trained lay counselor following WHO and Zambian national testing guidelines. Testing was conducted using Determine HIV-1/2 test (Abbott Laboratories, Abbott Park, USA) and confirmatory tests using Uni-Gold HIV test (Trinity Biotech, Bray, Ireland). Inmates who chose not to be tested for HIV continued with TB screening procedures. Inmates were then referred to a mobile laboratory to receive digital chest radiography (CXR). On Day 2, inmates were reconvened for the CO to perform a physical examination, review TB risk factors, symptoms, and smear results, interpret the CXR, and make a determination on the diagnosis of TB.

Exit screening was conducted *prior* to other activities to evaluate TB prevalence in inmates without the influence of the screening intervention. The smaller sample size in this group enabled both sputum specimens to cultured. However, because of the early timing, the CXR unit was not yet available and inmates were not evaluated for *clinical* TB. In addition, HIV test kits and counselors were not yet available for the majority of inmates going through exit screening and thus they were not offered PITC. All other procedures were the same as in the mass and entry screening protocol.

During community screening we used a door-to-door strategy to cover the entire prison camp. While screening was available for all individuals, budget constraints resulted in sputum samples being collected only from individuals presenting with TB symptoms and/or an abnormal CXR. As with the entry and mass screenings, only one of the two sputum samples was sent for culturing. PITC was offered as described in entry and mass screening.

### Laboratory Procedures

Sputum smears were examined and digital CXRs (EasyDR, Oldelft Benelux BV, NL) taken in an on-site semi-mobile 20-foot container, custom fitted as a digital X-ray/smear microscopy unit. All inmates had LED fluorescence microscopy (FM) (Primo Star iLED™Carl Zeiss Microimaging, Oberkochen, Germany) performed on two spot sputa. The highest quality specimen was then transported the same day under controlled temperature conditions to the TB department of the CIDRZ Central Laboratory, a BSL3 facility, for culturing. One sputum per inmate was cultured using both liquid (BD BACTEC™ MGIT™ 960 Mycobacteria Testing System) and solid (BD BBL™ Lowenstein-Jensen Medium) culture. *M. tuberculosis complex* (MTBC) speciation and drug susceptibility testing were done using line probe assay (GenoType MDR™, Hain Life Science GmbH, Germany). Support was provided by the ZAMBART laboratory when extra capacity was required.

### TB Diagnosis

A TB case was defined as either bacteriologically confirmed or clinically diagnosed. Bacteriologically confirmed cases were FM smear and/or culture positive. Clinically diagnosed cases were FM smear and culture negative, but had a CXR and/or signs and symptoms that were considered clinically consistent with TB by a trained CO. A patient was classified as ‘symptomatic’ if they had any of cough, fever, night sweats or weight-loss. Extra-pulmonary TB was diagnosed by a CO based on signs, symptoms and physical exam findings.

### Treatment of Diagnosed Cases

Patients diagnosed with TB were referred for initiation of anti-tuberculosis therapy (ATT) at an MOH TB treatment center adjacent to the prison facility. Patients *not* initially diagnosed with TB, but later found culture positive for MTBC were referred for ATT initiation upon receipt of culture results. Inmates who had been discharged before culture results were received were traced by community workers where possible.

Per national guidelines, patients found HIV-positive (regardless of TB diagnosis) were referred for enrollment into HIV care and treatment at the closest MOH HIV treatment center located two kilometers from the prison facility.

### Data Collection and Analysis

All data for this study were collected using standardized forms. An onsite study coordinator provided the first level of quality assurance by reviewing all files for completeness and plausible responses and resolving apparent issues in real time. Following screening, all forms were entered into a custom Microsoft Access database by four data-entry clerks. FM smear results were obtained daily from the microscopy laboratory register and culture results were generated by the CIDRZ CLTB information system in real-time as they became available. All data were exported into SAS 9.2 (Cary, North Carolina, USA) for subsequent cleaning and analysis. TB and HIV prevalence were calculated as proportions and 95% confidence intervals. We used chi-squared tests to compare (a) the prevalence of TB and HIV in different screening groups and (b) history of prior incarceration among inmates with and without TB and HIV. Requests for use of the raw data supporting our results should be directed to the Zambian Ministry of Home Affairs, Director of Prisons Health.

## Results

Between November 2010 and April 2011, a total of 2,514 participants were screened at Lusaka Central Prison during entry, mass, exit, and community screening. The average static inmate population during that period was 1,300, however high inmate turnover, particularly amongst remandees, contributed to our screening almost double that number.

Of the 2,514 participants screened, 2,323 had complete screening data and were included in the analysis. Screening participants were 88% male, with a median age of 31 and median body mass index (BMI) of 21.9. More than half (62%) of all those screened had one or more of the WHO-recommended screening symptoms of cough, fever, weight loss or night sweats, with the most commonly recorded symptoms being cough (43%) and weight loss (31%). Characteristics of screening participants are outlined in Table 2.

**Table 2:** Population Characteristics.

	Screening Intervention				
Characteristic	Entry (N = 371)	Mass (N = 1362)	Exit (N = 188)	Community (N = 402)	Total (N = 2323)
Sex					
Male, N (%)	368 (99.2%)	1293 (94.9%)	169 (89.9%)	205 (51.0%)	2035 (87.6%)
Female, N (%)	3 (0.8%)	69 (5.1%)	19 (10.1%)	197 (49.0%)	288 (12.4%)
Age, median (IQR)	28 (23–34)	32 (27–38)	32 (27–39)	25 (15–36)	31 (25–37)
History of TB					
Past, N (%)	26 (7.0%)	111 (8.1%)	34 (18.1%)	22 (5.5%)	193 (8.3%)
Current, N (%)	1 (0.3%)	33 (3.4%)	2 (1.1%)	3 (0.8%)	39 (1.7%)
Prior history of incarceration, N (%)	88 (23.7%)	315 (23.1%)	82 (43.6%)	32 (8.0%)	517 (22.3%)
Presented with any cough, fever, night sweats or weight loss	216 (58.2%)	825 (60.6%)	139 (73.9%)	250 (62.2%)	1430 (61.6%)

Table 3 presents results across all TB screening interventions. A total of 176 inmates (7.6%) were diagnosed with TB; 92% (n = 162) of whom where male. This did not include 52 persons (49 male, 3 female) already diagnosed and receiving TB treatment at the start of the program. The 176 cases consisted of 88 bacteriologically confirmed (3.8% of those screened) and 88 (3.8%) clinically diagnosed cases. Amongst the bacteriologically confirmed cases, 33% (n = 29) were asymptomatic and FM smear was positive in only 25% (n = 22). One of the culture confirmed cases (1.1%) had multi-drug resistant TB (MDR-TB). A history of incarceration was more common among inmates with bacteriologically confirmed TB (35.0%) than among inmates without TB (24.8%; p = 0.04).

**Table 3:** TB Prevalence.

Screening intervention	Total Screened	Already on ATT	Diagnosed at screening: Bacteriological confirmed^1^	Diagnosed at screening:Clinical diagnosis	All forms TB
Entry	371	1 (0.3%) [0.0–1.5%]	17 (4.6%) [2.7–7.2%]	9 (2.4%) [1.1–4.6%]	27 (7.3%) [4.9–10.4%]
Mass	1362	46 (3.4%) [2.5–4.5%]	53 (3.9%) [2.9–5.1%]	66 (4.9%) [3.8–6.1%]	165 (12.1%) [10.4–14.0%]
Exit	188	2 (1.1%) [0.1–3.8%]	10 (5.3%) [2.6–9.6%]	0 (0%)^2^ [0–0.2%]	12 (6.4%)^2^ [3.3–10.9%]
Community	402	3 (0.7%) [0.2–2.2%]	8 (2.0%)^3^ [0.9–3.9%]	13 (3.2%) [1.7–5.5%]	24 (6.0%) [3.9–8.8%]

1 Smear positive and/or culture positive for MTBC.

2 Chest x-ray and clinical work-up were not performed for the majority of inmates screened in exit screening; thus there were no clinical diagnoses; as a result, all forms TB was proportionally lower than in the other screening groups.

3 During community screening, only symptomatic patients (N = 184) had sputum collected for smear and culture.

*Entry* screening captured 371 inmates, representing 100% of inmates entering the facility during the intervention. Twenty-six cases of active TB were diagnosed (7.0%), with 17 (4.6%) bacteriologically confirmed and 9 (2.4%) clinically diagnosed. During *mass* screening, a total of 1,362 inmates were evaluated. TB was diagnosed in 119 (8.7%) inmates. Fifty-three (3.9%) were bacteriologically confirmed and 66 (4.9%) were clinically diagnosed. At *exit*, a total of 188 inmates were screened and 101 (5.3%) were diagnosed with TB; all of these cases were bacteriologically confirmed. There was no statistically significant difference in the prevalence of bacteriologically-confirmed TB between the entry, mass, and exit screening groups (p = 0.79). During *community* screening, 402 staff, family and community members were screened and 21 (5.2%) cases of TB were diagnosed: eight (2.0%) were bacteriologically confirmed, and 13 (3.2%) clinically diagnosed.

HIV testing results and the prevalence of co-infection are shown in Table 4. During *entry* screening 313 (84%) inmates had a known prior status (positive at any time in the past or negative result in the three months prior to screening) or accepted HIV testing, and 20.5% were HIV-positive. During mass screening, 1,247 (92%) had a known prior status or accepted testing with 27.4% found HIV-positive. The HIV prevalence during mass screening (27.4%) was significantly higher than in inmates entering prison (20.5%, p = 0.01). Due to limited staffing during the exit intervention, PITC was offered to only 35/188 inmates screened at *exit*. Twelve of these inmates (34.3%) were HIV positive. Because this is such a small sample of our exiting inmates, we did not compare them statistically to the other screening groups. A history of previous incarceration was marginally more common amongst HIV-positive inmates (27.3%) than amongst HIV-negative inmates (22.7%; p = 0.06). Uptake of testing was also lower (58%) during *community* screening, due to a higher refusal rate than in other groups. Of those tested, 25% were HIV-positive.

**Table 4:** HIV prevalence and TB/HIV co-infection.

Screening Intervention	Total Screened	Number with known HIV status^1^	HIV positive	Proportion of HIV+ persons with bacteriologically-confirmed TB	Proportion of HIV- persons with bacteriologically-confirmed TB	Proportion of bacteriologically –confirmed TB patients that are HIV+
Entry	371	313 (84%) [80–88%]	64 (20.5%) [16.1–25.4%]	5/64 (7.8%) [2.6–17.3%]	9/249 (3.6%) [1.7–6.8%]	5/14 (35.7%) [12.8–64.9%]
Mass	1362	1247 (92%) [90–93%]	342 (27.4%) [25.0–30.0%]	22/342 (6.4%) [4.1–9.6%]	26/905 (2.9%) [1.9–4.2%]	22/48 (45.8%) [31.4–60.8%]
Exit	188	35^2^ (19%) [13–25%]	12 (34.3%) [19.1–52.2%]	1/12 (8.3%) [0.2–38.5%]	1/23 (4.4%) [0.1–22.0%]	1/2 (50.0%) [1.3–98.7%]
Community	402	232 (58%)^3^ [53–63%]	57 (24.6%) [19.2–30.6%]	7/57 (12.3%) [5.1–23.7%]	0/175 (0%) [0.0–2.1%]	7/7 (100%) [59.0–100%]

1 Includes prior positives, persons who had tested negative within the 3 months prior to screening, and those who accepted PITC at screening.

2 Due to staffing limitations and unavailability of test kits at screening start-up, the majority of inmates participating in exit screening were not offered PITC.

3 The lower uptake of PITC in community screening is primarily due to persons declining HIV testing.

## Discussion

Our findings provide a critical first look at the joint burden of TB and HIV disease in a large sub-Saharan African prison facility. Although a number of prison studies have reported on either TB [12], [15]–[18] or HIV prevalence [13], [19]–[22], to our knowledge, no other study in sub-Saharan African prisons reports concurrently on baseline prevalence for *both* diseases. In view of the well-established biological and clinical linkages between TB and HIV, the high baseline rates of infection in sub-Saharan Africa and the acknowledged high-risk environment of prisons, these findings thus constitute an important contribution to the evidence base.

The prevalence of previously undiagnosed, bacteriologically confirmed TB among inmates residing in Lusaka Central Prison was 3900/100,000. This is 4.5 times the prevalence of bacteriologically confirmed TB found in the general population of Lusaka Province [9]. HIV prevalence among inmates residing within Lusaka Central Prison was found to be approximately twice that of the national prevalence (27% vs 13.5%) [10] and 30% greater than in Lusaka province (27% vs 21%) [11].

In Zambia, as elsewhere, individuals in poor health are likely to be overrepresented amongst those who enter the criminal justice system. This ‘concentration’ occurs in part because behavioral and structural factors that contribute to poor health (e.g. unemployment, illicit drug use, poverty and alcoholism) also contribute to the likelihood of incarceration. However, this concentration of disease amongst inmates entering the system fails to account for the increased prevalence of both TB and HIV disease seen between the mass and exit screening protocols. Such an increase suggests disease acquisition, not just concentration within the facility.

HIV prevalence increased from entry (20%) to mass (27%) screening, suggesting possible disease transmission within the prison (Table 4). The prevalence at exit is even higher (34%), but this figure should be interpreted with caution: it is based on a small sample of exiting inmates and has a very large confidence interval [19.1–52.2%]. Environmental conditions may promote HIV transmission, with overcrowding contributing to normalization of behaviors that facilitate disease-transmission such as violence and unprotected sex. Similarly, adverse environmental conditions such as poor nutrition, psychological stressors and limited access to testing and treatment may contribute to disease progression, thus increasing the risk of transmission when a person engages in risky behaviors. Meanwhile, access to preventive interventions, psycho-social and or other rehabilitative services remain currently weak or non-existent [23]–[25].

**Figure 1:**
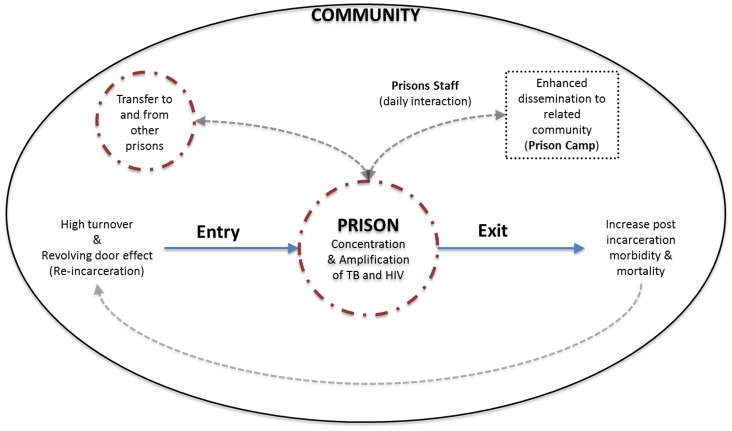
Theoretical model: Prisons – Community interaction. Connections with the outside community through released inmates and interaction with prisons staff result in disease dissemination to the outside community. The revolving door effect of re-incarceration further concentrates TB and HIV within the penitentiary system, increasing transmission within the prisons and to the outside community.

While the overall TB prevalence was lower at exit screening than at both entry and mass screening, this most likely reflects the lack of CXR which prevented clinical diagnoses. A potential explanation for the similar rates of TB at entry and mass screenings is that most inmates entering the prison came from police detention centers (jails) where they could have stayed anywhere from a day to several months. The detention centers have many environmental similarities to prisons and are probably high-risk environments for TB transmission. Another potential explanation is the ‘revolving door’ effect (Fig. 1). High rates of active TB in the prison contribute to higher-than-normal rates of latent TB infection (LTBI) among inmates. Continual inmate turnover, and high rates of re-imprisonment (Table 2) make it likely that re-offenders who were previously infected with LTBI, develop active disease prior to their subsequent prison tour. Conditions of extreme physical and emotional duress as well as high rates of HIV infection likely also contribute to this trend. This cycle of post-release morbidity, which in turn contributes to higher rates of disease amongst re-offenders, is supported by our finding of a significant association between prior imprisonment and bacteriologically confirmed TB, and a trend towards an association between prior imprisonment and HIV infection.

In addition to high rates of disease *within* the prison, the prevalence of bacteriologically confirmed TB in the community immediately surrounding the prison (2000/100,000) was more than twice the prevalence found previously in Lusaka province [9]. While the study design here precludes causal inference, this finding is suggestive of disease transmission between those working in the prison and the general community (Fig. 1). In Lusaka Central Prison, community contact occurred when inmates were released from prison, when prison officers returned daily to their families and friends outside the prison, and when inmates received visitors from the outside community. This hypothesis is supported by previous studies showing that the population attributable fraction (PAF) of TB in the general community due to exposure in prisons is substantial, with the median PAF among several studies in lower to middle income countries being 6.3% (IQR:2.7–17.2%) [26].

The finding of low sensitivity of smear microscopy (25%) in this study provides further evidence of the need for better and faster tools to diagnose TB. Technologies such as the Xpert® MTB/RIF assay [27], [28] are timely in this respect. Since 33% of the bacteriologically confirmed TB cases identified in this study did not report any of the typical screening symptoms of cough, fever, weight loss or night sweats, our findings point to the need for an algorithm based on different criteria to facilitate more aggressive screening, diagnosis and treatment of prison-based cases.

### Limitations

This study had several limitations. Due to operational constraints, there was no CXR during the exit screening protocol; this likely contributed to an underestimation of clinical disease in this population. Conversely, the necessarily aggressive approach to TB diagnosis in prison settings may have resulted in over-diagnosis of clinical cases during the entry and mass screening interventions. We sought to mitigate this by conducting training and refresher courses in radiographic interpretation, standardizing report forms and providing ongoing mentorship and quality control by a trained infectious disease physician. The few persons tested for HIV during exit screening reflected staff constraints and unavailability of test kits; whereas community members were less likely to accept HIV testing when offered. The lower acceptance of HIV testing in community members is likely due to stigma; persons were more accepting of TB screening because TB is less stigmatized than HIV.

Rates of bacteriologically confirmed TB may have been underestimated due to culturing only a single specimen per inmate during entry, mass and community screening. Ideally we would have cultured at least two specimens per inmate but this was cost prohibitive. As the primary goal of the program was case finding, we opted to screen a greater number of inmates with one specimen rather than fewer inmates with two specimens. Each specimen was cultured in solid and liquid media providing the minimum reference standard and minimizing the difference in yield between one and two specimens. In addition, time, space and security constraints meant we relied on trained inmate peer educators to assist in collection of sputum specimens during the entry and mass screening interventions. This resulted in a lack of consistency in specimen quality that may have affected TB yield, as suggested by the high proportion of inmates who were culture negative but with radiographic abnormalities consistent with TB.

## Conclusion

In this paper, we describe extremely high rates of TB and HIV in Lusaka Central Prison, and suggest mechanisms that may contribute to disease concentration, and transmission. Although confirming a widely held assumption, these findings constitute an alert not only to the poor health of prison inmates, but to the way poor prisoner health may threaten community disease control efforts. To tackle this dual burden of disease, a coordinated strategy among government institutions and stakeholders is urgently needed to implement legislative and criminal justice system reform, invest in institutional and health system upgrades and enable preventive measures within the prison environment.
